# Single-cell RNA-seq data analysis characterizing bronchoalveolar epithelial cells in patients with SARS-CoV-2 infection

**DOI:** 10.1186/s12950-022-00310-1

**Published:** 2022-09-05

**Authors:** Zhiqin Deng, Qin Li, Yongshen Li, Zhenhan Deng, Xiaoqiang Chen, Zhe Zhao, Guganghui Wang, Daping Wang, Jianquan Liu, Wencui Li

**Affiliations:** 1 Hand and Foot Surgery Department, Shenzhen Second People’s Hospital (The First Hospital Affiliated to Shenzhen University), 3002 Sungang West Road, Shenzhen, 518000 China; 2 Department of Laboratory Medicine, Guizhou Provincial People’s Hospital, Guiyang, 550002 China; 3 Department of Sports Medicine, Shenzhen Second People’s Hospital/ the First Affiliated Hospital of Shenzhen University Health Science Center, Shenzhen, 518000 Guangdong China; 4 Department of Biomedical Engineering, Southern University of Science and Technology, Shenzhen, 518055 China

**Keywords:** SARS-CoV-2, Alveolar epithelial cells, ACE2, scRNA-seq

## Abstract

**Background:**

Angiotensin-converting enzyme 2 (ACE2) has been reported to be the main receptor for SARS-CoV-2 infection of host cells. Understanding the changes in bronchoalveolar epithelial cells after SARS-CoV-2 infection of host cells and the intercellular communication relationship between these epithelial cell changes and immune cells is of great significance for the development of therapeutic methods.

**Methods:**

We explored the single-cell RNA sequence (scRNA-seq) of cells infected with bronchoalveolar lavage fluid (BaLF) of patients with different severities of SARS-CoV-2 and healthy people.

**Results:**

We found 11 clusters of epithelial cells in the BaLF, and they were derived from the S group. In the S group, the proportion of cells with positive ACE2 expression was relatively high. ACE2 was relatively more expressed in epithelial cell clusters 1, 3, and 7. Clusters 4 and 5 represented the original state, and there were two differentiation directions: one was cluster 2, and the others were clusters 1, 3, and 6. Cluster 7 was the intermediate state. Clusters 1, 3, 6, and 7 had high similarities (> 0.9), and their main signaling pathways focused on inflammatory activation and immune response. Cluster 2 was relatively specific and was up-regulated in differential genes that were mainly related to apoptosis. The ligand-receptor expression pattern of TNFRSF10D-TNFSF10 showed a special inter-cell regulatory relationship between epithelial cell cluster 2 and macrophages.

**Conclusion:**

This study revealed the changes in epithelial cells derived from alveolar lavage fluid after SARS-CoV-2 infection and the communication relationship with other immune cells.

## Introduction

At present, novel coronavirus is widespread globally and has caused serious health, economic, and social problems. Experts and scientists around the world are rapidly working to expand scientific knowledge about the new virus. The primary clinical symptoms of SARS-CoV-2 include cough, fever, and severe pulmonary infection, followed by sputum production and fatigue [1]. The first step of SARS-CoV-2 infection is that the virus enters human cells. These coronaviruses have a similar spike protein three-dimensional structure and are thought to have a strong binding affinity with the human cell receptor angiotensin-converting enzyme 2 (ACE2). Human ACE2 is the docking and entry receptor of severe acute respiratory syndrome coronavirus (SARS-CoV) in human cells [2, 3] and may also be the cell receptor of a new coronavirus (SARS-CoV-2) [4]. It plays an important role in the process of cell entry into the human body [5]. ACE2-expressing cells may serve as target cells and may be susceptible to SARS-CoV-2 infection, as reported in literature [6]. Therefore, organs with a high expression of ACE2 should be considered as organs with potential high risk for SARS-CoV-2 infection. ACE2 is expressed in bronchial epithelial cells, oral epithelial cells, absorbent intestinal cells of ileum and colon, hepatobiliary duct cells, and proximal renal tubule cells [6, 7].

To investigate the potential pathway of novel coronavirus to infect epithelial cells in alveolar lavage fluid, we used the dataset GSE145926 from the comprehensive database Omnibus [Gene Expression Omnibus (GEO)]. Single-cell sequencing samples were included from tracheoalveolar lavage fluid from patients with different degrees of SARS-CoV-2 infection and healthy individuals. Through a series of analyses, we explored the expression of ACE2 in alveolar lavage fluid epithelial cells and the composition and proportion of cells expressing ACE2. By comparing the genetic characteristics and functional differences of epithelial cells, and the cell communication relationship with immune cells, we explored the potential molecular mechanism of the onset of SARS-CoV-2 and provided clues about possible infection pathways.

## Materials and methods

### Microarray data

The National Center for Gene Expression Synthesis of Biotechnology Information (NCBI GEO) is a public online resource repository for high-throughput gene queries and high-throughput gene expression tests for the global research community [8]. This study used this method to download scRNA-seq data of patients with SARS-CoV-2 infection. The gene expression profile of GSE145926 was downloaded from the GEO database and included three alveolar lavage cell samples from patients with moderate SARS-CoV-2 (M1–M3), six alveolar lavage cell samples from patients with severe/critical SARS-CoV-2 infection (S1–S6), three healthy controls (HC1–HC3), and one open BaLF (HC4) sample dataset, which was downloaded from the GEO GSE128033 database [9]. Moderate and severe / critical disease the detailed classification and definition criteria are shown in the reference 9.

### Single-cell data dimensionality reduction and cluster analysis

The principal component (PCA) linear dimensionality reduction analysis utilized gene expression. Non-linear dimensionality reduction (tSNE) visualized the PCA results in a two-dimensional space.

### Differential gene screening and signal pathway enrichment analysis

Differential gene screening used the function FindMarkers, which is included in the Seurat package. The screening condition for differentially expressed genes (DEGs) was |log2FoldChange|> 1.5 and *P* < 0.05. The GO and KEGG enrichment analyses of significantly different genes were performed by the hypergeometric distribution test.

### Pseudotime analysis

The development pseudo-time was completed using Monocle2 [10]. The import CDS function included in the Monocle package was used to convert the Seurat object into a Cell DataSet object for raw counting. We selected sorting genes (qval < 0.01) using the differential Gene Test function. The dimensional reduction clustering analysis was performed using the reduce Dimension function, and then, the trajectory inference used the order cells function with default parameters. Finally, we used the function plot genes in pseudotime to draw gene expression to track changes in pseudo-time [11].

## Results

### Epithelial cell clusters in bronchoalveolar lavage fluid in patients with SARS-CoV-2 disease

**Fig. 1:**
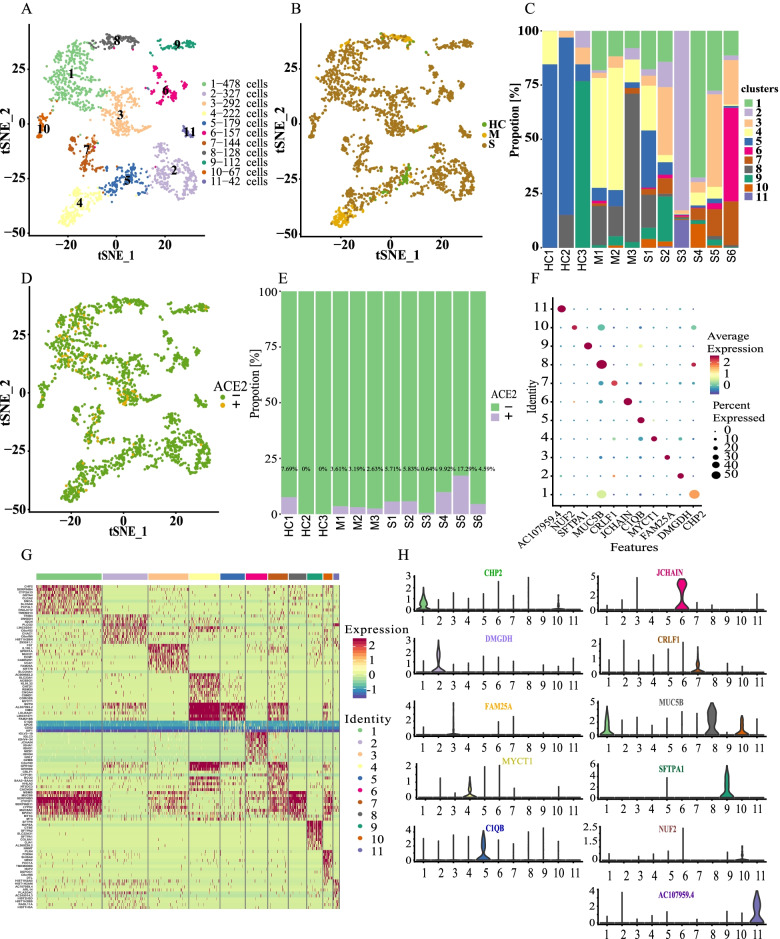
Epithelial cell clusters in bronchoalveolar lavage fluid in patients with SARS-CoV-2 disease. **A** The 11 BaLF epithelial cells types. **B** Distribution of 11 BaLF epithelial cells types in healthy controls (HC) and patients with moderate (M) and severe (S) SARS-CoV-2 infection. **C** Component of epithelial cells in each sample. **D** Expression of ACE2 in epithelial cells in each group. **E** Histogram of ACE2 gene expression in epithelial cells. **F** Top marker gene dot plot of clusters 1–11. **G** Heat map of differential genes in cell-type clusters. **H** Violin plots showing distribution of expression for selected cluster marker genes.

The epithelial cells in the BaLF samples from the M group of moderately infected SARS-CoV-2 patients, the S group of severely infected SARS-CoV-2 patients, and the control group (HC) of healthy people were divided into groups. The differences between the epithelial cells in the three groups were compared. The results showed that all epithelial cells were divided into 11 sub-clusters (Fig. 1A). The distribution of epithelial cells in the three groups (HC, M, and S) showed that epithelial cells were basically derived from the lavage fluid of Group S, while there were few epithelial cells in the lavage fluid of Group HC and Group M (Fig. 1B). The distribution of subsets of epithelial cells in the 13 samples is shown in Fig. 1C. The distribution of cells in the epithelial subsets of each sample and the corresponding cell numbers are detailed in Table 1. It can be seen that with the progression of SARS-CoV-2, the number of shedding epithelial cells in the alveolar lavage fluid increased. The situation of epithelial cells was further explored, and the expression of ACE2 gene in these epithelial cells was demonstrated. The results showed that ACE2 had relatively high expression in clusters 1, 3, and 7, and a small expression in clusters 4 and 5 (Fig. 1D). The histogram of the proportion of ACE2 gene expression in epithelial cells (Fig. 1E) shows that there were certain differences in the number, clustering, and positive rate of ACE2 expression between group M and group S in epithelial cells. In group S, the proportion of ACE2 positive cells was relatively high, which was consistent with the argument that ACE2 expression was the target of virus attack. To further understand the gene and functional characteristics of each cluster, we first used dot plot and violin plots to display the average expression and density distribution of the top1marker gene in the single-cell dataset clusters 1–11 of the cell-type cluster (Figs. 1F and H). In addition, a heat map was constructed between clusters 1–11 and the gene expression modules with clear differences (Fig. 1G). Among them, clusters 1, 3, 4, 5, and 7 highly expressed CHP2, ECM1, SLC23 A1, C1QB, and CRLF1. Clusters 2 and 6 highly expressed TRIB3 and IGLV3-19, and clusters 8, 9, 10, and 11 highly expressed MUC5B, SFTPA1, NUF2, and AC107959.4.

**Table 1:** The number, cluster distribution and proportion of epithelial cells

Group	Sample	Cell Number	Top1 Cluster and number of cells
HC	HC1	13	cluster5, 11
	HC2	33	cluster5, 27
	HC3	13	cluster9, 10
	HC4	0	
M	M1	83	cluster4, 42
	M2	94	cluster4, 53
	M3	38	cluster8, 26
S	S1	368	cluster5, 97
	S2	309	cluster3, 97
	S3	312	cluster2, 258
	S4	373	cluster1, 252
	S5	185	cluster3, 79
	S6	327	cluster6, 141

### Different functions of epithelial cells in patients with moderate and severe SARS-CoV-2 infection

**Fig. 2:**
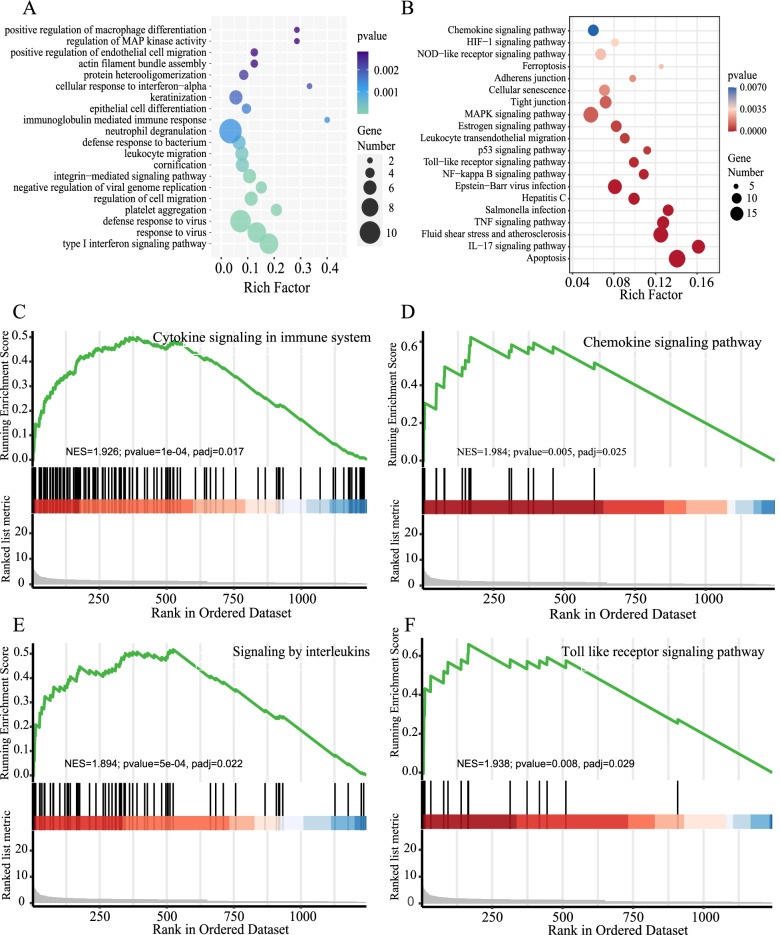
Differences in the function of epithelial cells in patients with moderate and severe SARS-CoV-2 infection. **A** Gene Ontology (GO) enrichment analysis of the up-regulated genes in group S vs. group M. The size of the black spots indicates the gene number. **B** The KEGG of the up-regulated genes in group S vs. group M. The color gradient represents the P value, and the size of the dots represents the gene number. Gene Set Enrichment Analysis (GSEA) of the up-regulated genes in group S vs. group M, such as cytokine signaling in immune system (**C**), the chemokine signaling pathway (**D**), signaling by interleukin (**E**), and Toll receptor signaling pathway (**F**)

To further understand the differences in epithelial cells between group S and group M, difference analysis and enrichment analysis of the corresponding gene function were performed on the epithelial cells of group S and group M. The GO functional enrichment analysis showed that the up-regulated genes in group S were mainly related to the regulation of cell migration, defense response to the virus, and response to the virus and type I interferon signaling pathway (Fig. 2A). The KEGG signaling pathway analysis results showed that the up-regulated genes in the S group were mainly enriched in the apoptosis, IL-17 signaling pathway, fluid sheer stress atherosclerosis, and TNF signaling pathway (Fig. 2B). Both GO and KEGG showed that the epithelial cells in group S were inflammatory.

GSEA enrichment analysis was performed on the epithelial cells of S and M groups. Similarly, it was found that cytokine signaling in the immune system, signaling by interleukins, the chemokine signaling pathway and Toll receptor, and other signaling pathways were enriched. Most signaling pathways were closely related to inflammation activation (Fig. 2 C–F).

### Interrelationships and functional changes among clusters of epithelial cell types expressing ACE2

**Fig. 3:**
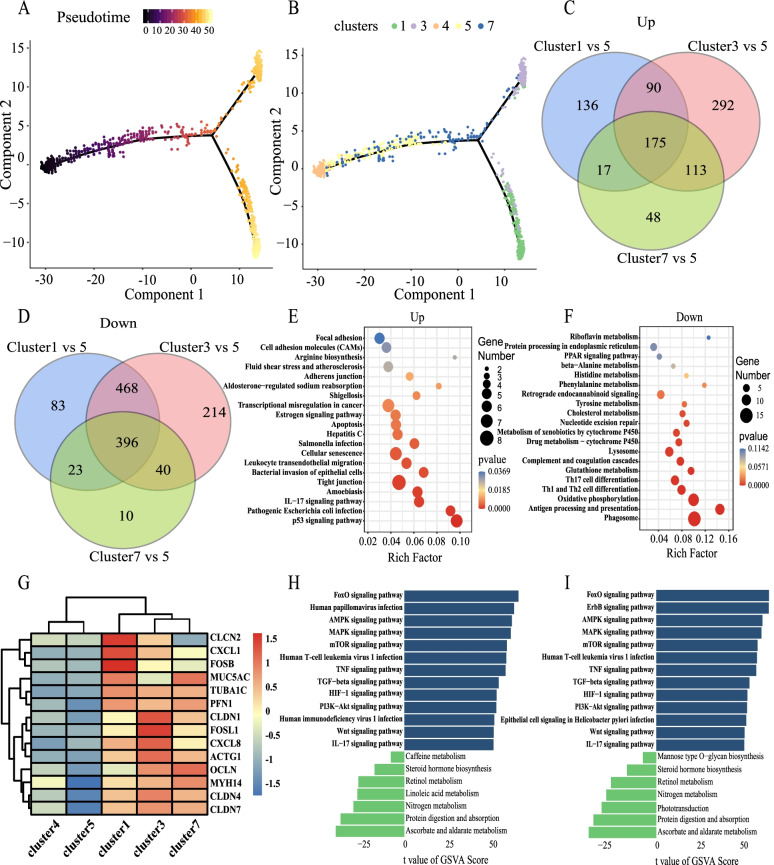
Interrelationships and functional changes among clusters of epithelial cell types expressing ACE2. **A** The pseudo-time trajectory of epithelial cell clusters 1, 3, 4, 5, and 7. Color from dark to light, indicating the differentiation time from early to late. **B** Pseudo-time trajectory of cell population differentiation in epithelial cell clusters 1, 3, 4, 5, and 7, which could be used to infer the differentiation relationships among cell populations at the initial step. **C** Venn diagram showing that the number of differences in cluster 7 vs. 5 was 48 up-regulated genes. **D** Venn diagram showing 10 down-regulated genes in cluster 7 vs*.* 5. **E** KEGG analysis of the Venn diagram intersection of 48 up-regulated genes. **F** KEGG analysis of the Venn diagram intersection of 10 down-regulated genes. **G** Gene heat map analysis of clusters 1, 3, 4, 5, and 7. Red indicates higher gene expression. Blue indicates lower gene expression. **H** GSVA bar chart of cluster 1 vs*.* cluster 5. **I** GSVA bar chart of cluster 3 vs*.* cluster 5. Green indicates no significant difference. Blue indicates significant difference. A T value greater than 0 denotes an up-regulated pathway, while a T value less than 0 denotes a down-regulated pathway

Owing to the relatively high expression of ACE2 in clusters 1, 3, and 7 and a small amount in clusters 4 and 5, the relationship among clusters 1, 3, 7, 4, and 5 cell populations with ACE2 expression was explored by the pseudo-time analysis method. The results showed that clusters 4 and 5 were ‘differentiated’ gradually through 7 to 1 and 3, suggesting that the epithelial cell populations of 7, 1, and 3, which were mainly concentrated with group S and expressed ACE, might have been attacked by the virus and subsequently changed, and 7 was an intermediate transition state (Fig. 3 A, B). This indicates that the SARS-CoV-2 virus may first recognize the ACE2 of the epithelial-cell-type clusters, clusters 4 and 5, and may then transform the epithelial-cell-type clusters into clusters 7, 1, and 3.

Because cluster 5 was the main cell population in the HC group and was in the early stage of the pseudo-time analysis, while clusters 1, 3, and 7 were mainly in group S, the Venn difference analysis method was used to consider cluster 5 as the control. Clusters 1 and 3 and 7 epithelial subsets and cluster 5 were compared with each other. The Venn diagram showed that the number of differences between clusters 7 and 5 was relatively small, with 48 up-regulated genes (Fig. 3 C) and 10 down-regulated genes (Fig. 3 D). Cluster 7 was an intermediate transition state, and the number of differences was relatively small. Venn diagram intersection of up-regulated KEGG genes was mainly concentrated in the p53 signaling pathway, pathogenic escherichia coli infection, and IL-17 signaling pathway (Fig. 3 E); Venn diagram intersection of down-regulated KEGG genes was mainly concentrated on phagosome, antigen processing and presentation, and oxidative phosphorylation (Fig. 3 F). The results showed that clusters 1, 3, and 7 not only showed inflammation but also a certain degree of apoptosis.

In addition, we selected genes related to inflammation and compared the differences between clusters 1, 3, 4, 5, and 7 by drawing heat maps. The results showed that inflammatory genes were significantly expressed in clusters 1, 3, and 7, which indicated the activation of inflammatory pathways. The high expression of MUC5AC also indicated the increase of mucus secretion (PMID: 30352166, PMID: 30352166). However, the expression of inflammatory genes was lower in clusters 4 and 5 (Fig. 3 G).

To further confirm that clusters 1 and 3 have the common characteristics of activating inflammatory pathways, GSVA analysis was conducted on clusters 1 and 3. Compared with cluster 5, the results showed that cluster 1 was mainly enriched in the FoxO signaling pathway, human papillomavirus infection, TNF, and IL-17 signaling pathway (Fig. 3 H). Similarly, compared with cluster 5, cluster 3 was mainly enriched in the FoxO signaling pathway, ErbB signaling pathway, AMPK, TNF, and IL-17 signaling pathway, among others (Fig. 3 I). Clusters 1 and 3 showed activation of inflammatory pathways.

### Functional changes of clusters of epithelial-cell types that did not express ACE2 after infection with SARS-CoV-2

**Fig. 4:**
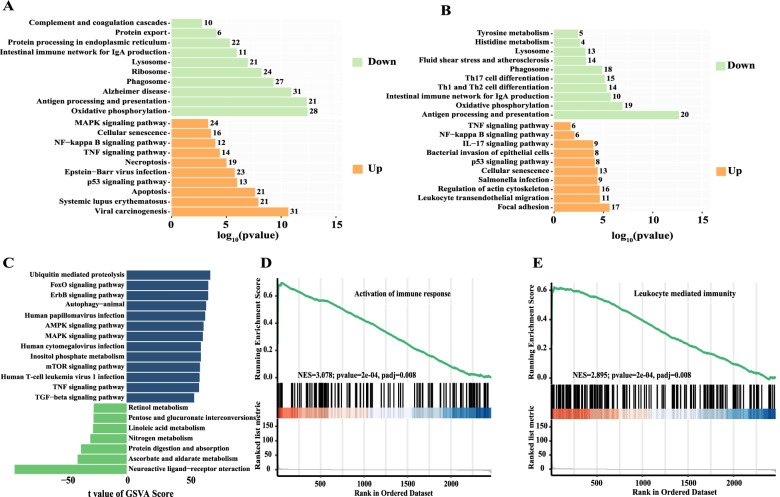
Functional changes of clusters of epithelial cell types for which no clusters express ACE2 after infection with SARS-CoV-2. **A** KEGG enrichment analysis of differential genes of cluster 2 vs*.* 5. **B** KEGG enrichment analysis of differential genes of cluster 6 vs*.* 5. **C** GSVA bar chart of cluster 2 vs*.* 5. GSEA of the up-regulated genes in cluster 6 vs*.* 5 such as activation of immune response (**D**) and leukocyte-mediated immunity (**E**)

The expression of clusters 2 and 6 was concentrated in group S, but the expression data showed that there was almost no expression of ACE2 in these clusters. To understand the change of clusters 2 and 6, KEGG enrichment analysis of differential genes of clusters 2 and 6 was conducted (Top 10). The results showed that, compared with cluster 5, many up-regulated genes of clusters 2 and 6 were related to inflammation, suggesting that clusters 2 and 6 might be related to clusters 1, 3, and 7, which might also be affected by the virus. Cluster 2 showed a strong apoptotic function, while cluster 6 did not show that ability (Fig. 4 A, B).

To further understand the relationship between clusters 2 and 6 and 1, 3, 7, 4, and 5, the relationship between these pathways was further studied. GSVA analysis showed that, compared with cluster 5, cluster 2 was mainly enriched in ubiquitin-mediated proteolysis, the TGF-beta signaling pathway, the TNF signaling pathway, and the human cell leukemia virus 1 infection signaling pathway (Fig. 4 C)—while, compared with cluster 5 epithelial cells, cluster 6 epithelial cells were enriched in the active immune response and leukocyte-mediated immunity signaling pathway (Fig. 4 D, E). Both the GSVA analysis and GSEA results of clusters 2 and 6 showed activation of inflammation and immune function after infection with SARS-CoV-2.

### Interaction between clusters of different epithelial cell types after SARS-CoV-2 infection

**Fig. 5:**
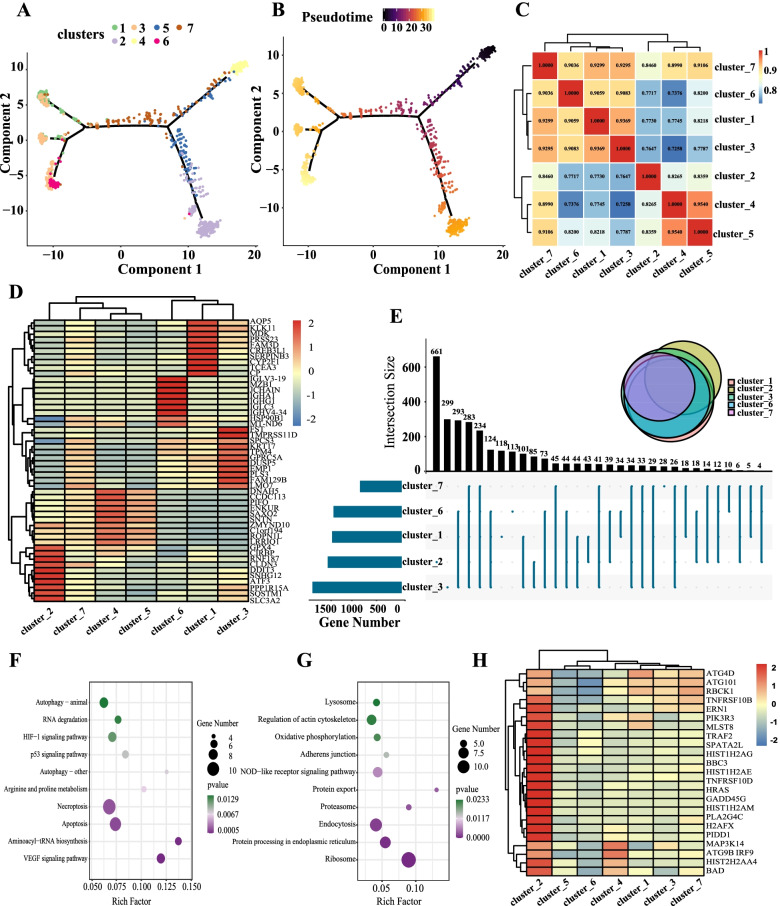
Interaction between clusters of different epithelial cell types after SARS-CoV-2 infection. **A** The pseudo-time trajectory of epithelial cell clusters 1, 2, 3, 4, 5, 6, and 7. Color from dark to light, indicating the differentiation time from early to late. **B** Pseudo-time trajectory of cell population differentiation in epithelial cell clusters 1–7. **C** Similarity heat map between clusters 1–7. Similarity heat map between clusters 1–7. Red represents high similarity, and blue represents low similarity. **D** Fifty marker genes of clusters 1–7 were selected for heat mapping display. **E** UpSet diagram of the differentially expressed genes in clusters 1, 3, 7, 2, and 6. **F** KEGG signaling pathway analysis of up-regulated differential genes in epithelial cell cluster 2. **G** KEGG signaling pathway analysis of down-regulated differential genes in epithelial cell cluster 2. **H** Heat map of cell death gene expression module in epithelial cell clusters 1–7

The previous results showed that clusters 2 and 6 were mainly found in group S, and the functions of clusters 2 and 6 also showed activation of immunity and inflammation. Thus, was there a certain degree of difference between cluster 2 and cluster 6, compared with clusters 1, 3, and 7? To find out, clusters 1, 3, 7, 2, 6, 4, and 5 were put together for the pseudo-time analysis (the relationships among 1, 3, 7, 4, and 5 are shown in Fig. 3). The results showed that clusters 4 and 5 were in the original state and differentiated in two directions—one was cluster 2, and the other included clusters 1, 3, and 6. Cluster 7 was an intermediate state (Fig. 5 A, B).

To further determine the relationship between clusters 1, 2, 3, 4, 5, 6, and 7, similarity heat map analysis was conducted on these clusters. The results showed that clusters 1, 3, 6, and 7 had relatively high similarity (> 0.9), and clusters 4 and 5 were similar (similarity > 0.9). Cluster 2 was relatively specific, and the differences between cluster 2 and the other clusters were relatively large. This result was consistent with that of pseudo-time analysis. This suggested that although cluster 2 was also activated by inflammatory pathways compared with cluster 5, this cluster might have had its own special characteristics (Fig. 5 C). Fifty marker genes of clusters 1–7 were selected for mapping display. The results also showed a certain degree of similarity between clusters 1, 3, and 6, and cluster 2 had specificity (Fig. 5 D).

The results of the pseudo-time analysis showed that cluster 7 was a cell population that transitioned to clusters 1, 3, and 6, and cluster 6 was similar to clusters 1 and 3. Cluster 2 was another branch. Comparing the differences between clusters 1, 3, 7, 2, and 6 with cluster 5, the UpSet results showed that the differential genes of cluster 7 were indeed relatively few. Moreover, the intersection of the differential genes among several groups was strong, and cluster 2 had a large number of differential genes with specific changes (Fig. 5 E). This suggested that cluster 2 may have been different from the other clusters to some extent (and in the results of pseudo-time analysis, cluster 2 was also independently divided into one cluster, Fig. 5 A, B).

To clarify the functional characteristics of cluster 2, the specific differential genes of cluster 2 were differentiated, and the corresponding functional enrichment analysis was conducted. KEGG signaling pathway analysis results showed that the up-regulated differential genes of cluster 2 were mainly related to the VEGF signaling pathway, apoptosis, and necroptosis (Fig. 5 F). The down-regulated differential genes of cluster 2 were mainly enriched in the ribosome, protein processing in the endoplasmic reticulum, and endocytosis (Fig. 5G). The single-cell dataset clusters 1–7 of the cell-type cluster and the cell death gene expression module were used to construct a heat map. The results showed that many apoptotic genes in cluster 2 began to be highly expressed (such as BAD and TNFRSF10B), indicating that the apoptotic condition of cluster 2 was significantly higher than that of other clusters (the genes of clusters 1, 3, 7, and 4 were relatively higher than those of clusters 5 and 6) (Fig. 5 H).

### The communication relationship between epithelial cell-type clusters and immune cells

**Fig. 6:**
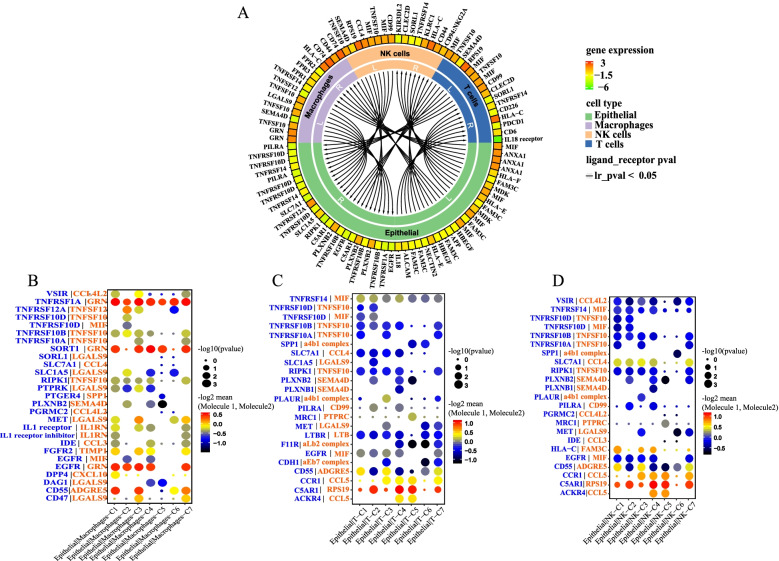
The communication relationship between epithelial cell-type clusters and immune cells. **A** Circos diagram of cluster 2 and immune cells. The solid line in the figure indicates that the interaction relationship is significant. The first circle represents the expression level of the ligand/receptor gene; the higher the red expression level, the lower the green expression level. The second circle represents cell types, such as epithelial cell cluster 2, macrophages, T cells, and NK cells. The third circle represents the ligand (L)/receptor (R) to which the gene belongs, and the fourth circle represents the interaction relationship between the ligand-receptor gene pair. **B** Cell communication dot diagrams between clusters 1–7 and macrophages. **C** Cell communication dot diagrams between clusters 1–7 and T cells. **D** Cell communication dot diagrams between clusters 1–7 and NK cells. The row represents the ligand-receptor pair that has a communication relationship between cells. The column represents the cell type (receptor cell | ligand cell) where cell communication occurs. The red color of the circle represents the expression value of the interacting cell gene, that is, the redder the circle, the greater the expression value

To understand the signal communication relationship between epithelial cells and immune cells, we used single-cell RNA sequencing data, took the gene expression data of cell subpopulations as the research object, used the ligand-receptor database, and used cellphoneDB to obtain information about ligands and receptors in cells to reflect the interactions between cells [12]. Figure 6 shows the communication relationship between the cluster 2 epithelial cells and the T, NK, and macrophages. Among them, MIF was highly expressed in the cluster 2 epithelial cells, and its receptor TNFSRSF14 was expressed in macrophages. The MIF secreted by the cluster 2 epithelial cells could interact with the receptor TNFSRSF14 in macrophages. In addition, the GRN secreted by macrophages could interact with the EGFR receptor of the cluster 2 epithelial cells. The expression pattern of the MIF-TNFSRSF14 and GRN-EGFR ligand-receptor complex interaction could accurately describe the interaction between the cluster 2 epithelial cells and macrophages. Similarly, the interaction between NK cells and cluster 2 epithelial cells, T cells, and cluster 2 epithelial cells could be accurately reflected (Fig. 6 A).

Furthermore, we compared the communication relationship between different clusters of epithelial cells and macrophages, T cells, and NK cells. Similar findings, the ligand-receptor expression patterns of TNFRSF10D-TNFSF10, TNFRSF10D-MIF, and TNFRSF10B-TNFSF10, showed that the cluster 2 epithelial cell cluster was different from other clusters and had a special interaction with macrophages but not with T cells and NK cells (Fig. 6 B, C, D).

## Discussion

Coronavirus infections have caused multiple outbreaks, including human respiratory tract infections, such as severe acute respiratory syndrome (SARS) in 2002 and middle east respiratory syndrome (MERS) in 2012. In December 2019, a novel corona virus (SARS-CoV-2) caused a pneumonia outbreak in Wuhan, China, again posing a public health risk from coronavirus [13]. The pathway and pathogenesis of this corona virus infection are still not fully understood; so, it is of great significance to deepen our knowledge of it. Although healthcare professionals and researchers worldwide are working hard to do so, thus far, the WHO has reported that there have been more than 4 million deaths worldwide due to SARS-CoV-2. One of the main methods of transmission of the new coronavirus is through inhaling the virus into the lungs, which causes inflammation of the lungs. However, its infection route and pathogenesis are not yet fully understood. Here, we focused on the changes in lung epithelial cells after virus infection, as well as the interaction between epithelial cells and other immune cells.

In this study, data from the public Bulk-Seq RNA dataset of two groups of patients with SARS-CoV-2 infection of different severities and a control group were analyzed. We found that there were relatively more epithelial cells in the lavage fluid of the severely infected group and a relatively high proportion of cells with positive ACE2 expression in the epithelial cells. Organs with high expression of ACE2 should be considered as potentially high-risk organs for novel coronavirus infection [6]. A large number of reports note the effects of ACE2 as the main receptor of SARS-CoV-2 infected cells on the renin-angiotensin system and other systems [14, 15]. It has been confirmed that ACE2 is mainly expressed in type II alveolar cells, hepatic bile duct cells, colonic epithelial cells, esophageal keratinocytes, ileal endothelial cells, rectal endothelial cells, gastric epithelial cells, and proximal tubules of the kidney and embryonic primordial germ cells [16–18]. These cells expressing ACE2 are also the main source of cells attacked by SARS-COV-2. We similarly found that CHP2, ECM1, SLC23 A1, C1QB, CRLF1, and other genes were relatively highly expressed in epithelial cell clusters 1, 3, and 7, which highly expressed ACE2. Moreover, in the severe group (S), the up-regulated genes were mainly related to signaling pathways such as regulation of cell migration, defense response to virus, response to virus and the type I interferon signaling pathway, apoptosis, the IL-17 signaling pathway, fluid sheer stress atherosclerosis, and the TNF signaling pathway. These pathways strongly indicate that in the severe group of patients’ lung epithelial cells, they are all related to apoptosis and cell inflammation.

Here, through a pseudo-time analysis, we also described the relationship and functional changes of the seven lung epithelial cell clusters after being attacked by the virus. The epithelial cell-type clusters 4 and 5 expressed a small amount of ACE2, which may be the first target of SARS-CoV-2. After being infected with the virus, the epithelial cells turned to epithelial cell clusters 1 and 3, among which cluster 7 may be an intermediate transition subtype. Correspondingly, signaling pathways such as the p53 signaling pathway, pathogenic *Escherichia coli* infection, and the IL-17 signaling pathway were activated, and the cells began to strongly manifest as apoptosis and inflammation.

We also noticed that in clusters 2 and 6 of the epithelial cells that appeared in the severe group, ACE2 was almost not expressed. However, they also showed the activation of the TGF-β signaling pathway, TNF signaling pathway, human-cell leukemia virus 1 infection, and other signaling pathways. These pathways are closely related to the activation of inflammation and immune function. Through the pseudo-time analysis, we found that clusters 4 and 5 were the original state, and there were two differentiation directions—one was cluster 2, and the other included clusters 1, 3, and 6; cluster 7 was an intermediate state. The results of similarity analysis confirmed this. The similarity between clusters 1, 3, 6, and 7 was high (> 0.9), and clusters 4 and 5 were relatively similar, while cluster 2 was relatively independent.

The above results showed that although the group S had a lot of epithelial cells, there was a difference between different subsets. Among them, the inflammatory response of clusters 1, 3, and 6 was more similar, the response for clusters 4 and 5 was relatively normal, and cluster 2 was relatively independent. However, from the perspective of cell apoptosis, the apoptotic ability of clusters 5 and 6 was relatively weak, the apoptotic ability of cluster 2 was the strongest, and clusters 1, 3, 4, and 7 showed a certain degree of apoptosis. In addition, these cell-type clusters were different in gene expression, and previous reports also point out the effects of virus infection on T cells and macrophages [9, 19]. Therefore, do different clusters of epithelial cells also present different communication and interaction relationships with other cells? Is the communication relationship between the independent cluster 2 and other cells significantly different from other clusters? The results showed the communication relationship between cluster 2 epithelial cells and T cells, NK cells, and macrophages.

We found that clusters of different epithelial cell types and immune cells exhibited a strong intercellular communication relationship. Among them, the ligand MIF was highly expressed in cluster 2 epithelial cells, and its receptor TNFSRSF14 was expressed in macrophages. Macrophages highly expressed the ligand GRN, while the EGFR receptor was expressed in cluster 2 epithelial cells. The ligand-receptor expression pattern of MIF-TNFSRSF14 and GRN-EGFR allowed cluster 2 epithelial cells and macrophages to form an intercellular communication relationship. Unlike other clusters, the cluster 2 epithelial cells and macrophages, but not T cells and NK cells, had a special inter-cell regulatory relationship.

## Conclusion

**Fig. 7:**
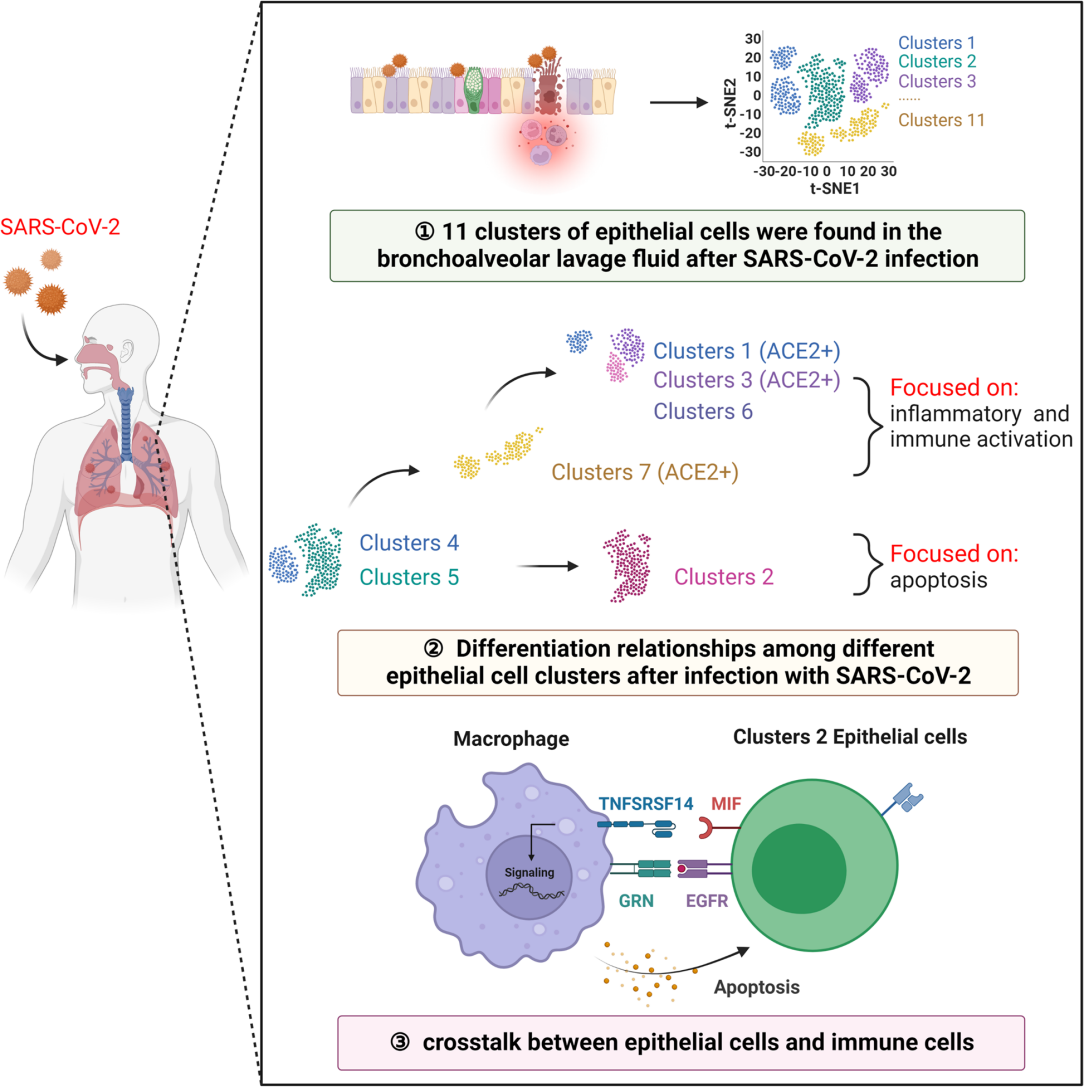
Characteristics of bronchoalveolar epithelial cells in patients with SARS-COV-2 infection. 11 clusters of epithelial cells were found in the bronchoalveolar lavage fluid after SARS-COV-2 infection. ACE2 was relatively more expressed in epithelial cell clusters 1, 3, and 7. There were two differentiation directions in Clusters 4 and 5: one was cluster 2, and the others were clusters 1, 3, and 6. The signaling pathways in Clusters 1, 3, 6, and 7 focused on inflammatory activation and immune response, and the Cluster 2 was related to apoptosis. The ligand-receptor expression pattern showed a special inter-cell regulatory relationship between epithelial cell and immune cells. Created with BioRender.com

This study revealed the changes of epithelial cells in bronchoalveolar lavage fluid after SARS-COV-2 infection and their communication relationship with other immune cells. Eleven clusters of epithelial cells were found in BaLF in severely and moderate infected patients. ACE2 was relatively more expressed in epithelial cell clusters 1, 3 and 7. Cluster 4 and cluster 5 represent the original state, and there are two differentiation directions, one is cluster 2, which is related to apoptosis. The other is clusters 1, 3, 6 and 7, associated with inflammatory activation and immune responses. The ligand-receptor expression patterns of TNFRSF10D-TNFSF10, TNFRSF10D-MIF, and TNFRSF10B-TNFSF10, among others, showed specific crosstalk between epithelial cell cluster 2 and immune cells (Fig. 7).

## Data Availability

The single-cell sequencing data of the SARS-CoV-2 patients in this project are from NCBI (https://www.ncbi.nlm.nih.gov/gds/), downloaded from GEO (GSE145926 and GSE128033).
